# Monkeypox Virus Neutralizing Antibodies at Six Months from Mpox Infection: Virologic Factors Associated with Poor Immunologic Response

**DOI:** 10.3390/v16050681

**Published:** 2024-04-26

**Authors:** Angelo Roberto Raccagni, Alessandro Mancon, Sara Diotallevi, Riccardo Lolatto, Elena Bruzzesi, Maria Rita Gismondo, Antonella Castagna, Davide Mileto, Silvia Nozza

**Affiliations:** 1 Infectious Diseases Unit, Vita-Salute San Raffaele University, 20132 Milan, Italy; bruzzesi.elena@hsr.it (E.B.); castagna.antonella1@hsr.it (A.C.); nozza.silvia@hsr.it (S.N.); 2 Laboratory of Clinical Microbiology, Virology and Bioemergencies, Ospedale Sacco, 20157 Milan, Italy; mancon.alessandro@asst-fbf-sacco.it (A.M.); mariarita.gismondo@unimi.it (M.R.G.); mileto.davide@asst-fbf-sacco.it (D.M.); 3 Infectious Diseases Unit, IRCCS San Raffaele Scientific Institute, 20132 Milan, Italy; diotallevi.sara@hsr.it (S.D.); lolatto.riccardo@hsr.it (R.L.); 4 Laboratory of Clinical Microbiology, Virology and Bioemergencies, University of Milan, 20157 Milan, Italy

**Keywords:** mpox, monkeypox, serology, immunity, neutralizing antibodies

## Abstract

A natural monkeypox virus infection may not induce sufficient neutralizing antibody responses in a subset of healthy individuals. The aim of this study was to evaluate monkeypox virus-neutralizing antibodies six months after infection and to assess the virological factors predictive of a poor immunological response. Antibodies were assessed using a plaque reduction neutralization test at six months from mpox infection; mpox cutaneous, oropharyngeal, and anal swabs, semen, and plasma samples were tested during infection. Overall, 95 people were included in the study; all developed detectable antibodies. People who were positive for the monkeypox virus for more days had higher levels of antibodies when considering all tested samples (*p* = 0.029) and all swabs (*p* = 0.005). Mpox cycle threshold values were not predictive of antibody titers. This study found that the overall days of monkeypox virus detection in the body, irrespective of the viral loads, were directly correlated with monkeypox virus neutralizing antibodies at six months after infection.

## 1. Introduction

The 2022 outbreak of mpox spread very rapidly worldwide, affecting disproportionately men who have sex with men (MSM) and people living with HIV (PLWH) [1,2,3,4]. In July 2022, the outbreak was determined to be a public health emergency of international concern (PHEIC) by the World Health Organization (WHO). The majority of people infected with mpox were young or middle-aged men, according to the reports of the Centers for Disease Control and Prevention (CDC). Sexual activity has been recognized early as the major risk factor for mpox infection, especially when considering members of specific key populations, such as MSM and PLWH. Infections were often self-limiting, although cases of hospitalization and death were reported. Antiviral treatment, including tecovirimat and cidofovir, was needed, particularly among immunodepressed people or PLWH with a low CD4^+^ T lymphocyte count [1,2,3,4]. The rapid dissemination of the mpox outbreak in 2022 triggered an international health emergency, spotlighting the virus’s capacity to breach geographical and societal boundaries with unprecedented velocity.

Before the current outbreak, natural infection was thought to provide lifelong immunity, as was known for smallpox. Few data are available regarding the immunological response over time to mpox infection, and to date, no data are available regarding the possible waning of immunity over years [5]. The investigation into the immune response elicited by mpox infection revealed a complex interplay between humoral and cell-mediated immunity. The rapid activation and differentiation of T cell populations following infection, along with the swift humoral response, highlighted the body’s multifaceted defense mechanism against the virus. Recent studies showed that monkeypox virus infection elicits both cell-mediated and humoral immunity in infected persons. As early as a few days after the onset of symptoms, CD4^+^ and CD8^+^naïve T cell populations rapidly decline in favor of terminally differentiated mpox-specific effector memory T cells, with the expression of immune markers [5]. Humoral response is equally rapid, with both IgM and IgG titers rising between 1 and 2 days after the onset and peaking, respectively, at 2 weeks and 2 months [6]. Mpox infection, because of the high cross-reactivity rate between orthopoxviruses, can lead to the generation of different subtypes of antibodies, such as anti-vaccinia virus (VACV) antibodies and mpox-specific neutralizing antibodies [7]. Moreover, mpox infection rapidly recalls anti-orthopox long-living humoral immunity if the host was previously vaccinated against smallpox [8].

While there is clear evidence of long-living humoral immunity against smallpox, induced by vaccination or a natural occurring disease that possibly lasts up to decades, there is still little evidence on the duration of immunity elicited by mpox [8,9,10]. Humoral response is much more crucial than cell-mediated immunity in guaranteeing protection against mpox infection [11].

Concerning mpox, previous outbreaks in endemic and non-endemic countries, along with the first couple months of the 2022 epidemic, were not defined by any case of viral reinfection, hence the hypothesis that mpox could not infect one a second time [12,13,14]. Strikingly, some individuals were later determined to have been possibly re-infected with mpox [14,15,16,17]. This new evidence opens up new questions regarding the durability and strength of mpox-induced protection following infection. One hypothesis for the occurrence of re-infections is that the monkeypox virus might not induce sufficient neutralizing antibody responses in a subset of healthy individuals. For instance, we previously described, at the Infectious Diseases Unit of San Raffaele Scientific Institute, two cases of mpox re-infection where mpox neutralizing antibodies were indeed detectable after the first episode of infection [16]. This likely suggests that mpox re-infection might also occur among people with evidence of detectable neutralizing antibodies after infection [16,17]. For instance, evasion strategies employed by the mpox virus to subvert immunological surveillance by virus-specific T cells are one contributing factor that might explain the virus's spreading abilities. While VACV and variola virus (VARV) have been extensively studied, monkeypox virus-specific strategies for evading immune detection and response remain relatively understudied [18,19].

To date, modified Vaccinia Ankara Bavarian Nordic (MVA-BN) (Bavarian Nordic A/S, Hellerup, Denmark), a third-generation vaccine based on a live, attenuated OPV, has been used during the current outbreak among members of key populations for infection control [1,2,3,4]. In our study setting, Italy, people recovering from mpox infection were not recommended to receive the MVA-BN vaccination.

From a clinical perspective, people diagnosed with mpox showed heterogeneous clinical manifestations, disease severity, and overall infection duration. Moreover, as previously reported, virologic analyses of different specimens also highlighted the complex and diversified viral dynamics of the monkeypox virus [20]. To date, little is known regarding whether these clinical and virologic characteristics have an influence on mpox immunologic and serologic dynamics.

The aims of this study were multifaceted and included both the evaluation of monkeypox virus neutralizing antibodies response six months after acute mpox infection and the comprehensive assessment of clinical and virologic factors contributing to a potential poor immunologic response. With monkeypox virus infections garnering increasing attention due to the potential for outbreaks and public health concerns, understanding the dynamics of the immune response appears to be paramount in assessing effective preventive and therapeutic strategies.

## 2. Materials and Methods

This is a retrospective sub-study including people with PCR-confirmed symptomatic monkeypox virus infection at the Infectious Diseases Unit of IRCCS San Raffaele Scientific Institute, Milan, Italy, between May and November 2023 [21]. People recently vaccinated against the monkeypox virus were excluded. People with suspected mpox infection were evaluated at the walk-in STI service of the Infectious Diseases Unit of IRCCS San Raffaele Scientific Institute, Milan, Italy, where mpox testing was conducted. Each person completed a questionnaire during the first visit regarding their previous clinical history and sexual behaviors at the time of diagnosis, which were recorded in the hospital electronic health records. Anal, oropharyngeal, and cutaneous swabs, plasma, and seminal fluid samples were collected every seven days circa until the end of infection and tested with monkeypox virus real-time polymerases chain reaction (RT-PCR), as previously described [21]. DNA was extracted from biological specimens using the QIA symphony DSP Virus/Pathogen Kit on the QIA symphony SP instrument (QIAGEN—Milan, Italy). A pan-Orthopoxvirus RT-PCR assay (RealStar^®^ Orthopoxvirus PCR Kit 1.0, altona DIAGNOSTICS—Milan, Italy), targeting variola virus and non-variola orthopoxviruses (cowpox, monkeypox, raccoonpox, camelpox, and vaccinia virus), was used to detect non-variola DNA; the diagnosis was confirmed with a monkeypox virus DNA-specific RT-PCR (Liferiver, Shanghai ZJ Bio-Tech Co.—Shanghai, China). Samples were deemed positive for monkeypox virus DNA with a cycle threshold (Ct) value ≤ 40.

Plaque reduction neutralization test (PRNT) was used to assess monkeypox virus-neutralizing antibody titers at six months from infection. More specifically, blood samples for antibody assessment were collected after a median time of 6.61 months (IQR = 6.17–7.22) from baseline. Blood samples were drawn (BD Vacutainer CAT serum collection tubes) and centrifuged for 10 min at 4 °C. Serum was collected, transferred into cryovials, and frozen at −80 °C. All tested serum samples were depleted by heat treatment (56 °C for 30 min). A total of 50 μL of serum, starting from a 1:10 dilution followed by a serial two-fold series, were transferred into wells of 96 weel microtiter plates (COSTAR, Corning Incorporated, Corning, NY, USA) and mixed with 50 μL of tissue culture infecting dose 50 (TCID 50) concentration of monkeypox virus (EPI_ISL_13302316). All dilutions were made in DMEM with 1% penicillin and streptomycin. After one-hour incubation at 37 °C and 5% CO_2_, 50 μL of 2 × 10^4^ VeroE6 (VERO-C1008-ATCC^®^-CRL-1586TM) cells were added to each well. After 6 days of incubation at 37 °C and 5% CO_2_, wells were stained with 0.1% crystal violet solution (Merck KGaA, 64271 Darmstadt, Germany) plus 5% formaldehyde 40% *m*/*v* (Carlo ErbaSpA, Arese, Milan, Italy) for 30 min; microtiter plates were washed in running water. Wells were scored to evaluate the degree of cytopathic effect (CPE) compared with the virus control; blue staining of wells indicated the presence of neutralizing antibodies. Neutralizing titer was the maximum dilution with a reduction of 90% of CPE; a positive titer was defined as ≥1:10. Positive and negative controls were included in all test runs, with every test including serum control (1:10 dilution), cell control (Vero E6 cells alone), and viral control (three-fold series dilution). Virologic and serologic analyses were performed at the Laboratory of Clinical Microbiology, Virology and Bioemergencies of Luigi Sacco University Hospital in Milan, Italy, which is a reference laboratory for orthopoxvirus testing. Recorded data were anonymized and managed according to Good Clinical Practice (GCP).

Individuals’ clinical and demographic characteristics were collected at each mpox-related visit and were retrieved from the Infectious Diseases Unit of the IRCCS San Raffaele Scientific Institute, Milan, Italy (Centro San Luigi [CSL] Cohort). The CSL cohort was approved by the Ethics Committee of the IRCCS San Raffaele Scientific Institute (4 December 2017, protocol n. 34). On their first visit, individuals provide written informed consent for the use of their anonymized data in scientific analyses. The conduct and reporting of this study were in line with the Declaration of Helsinki.

For the purpose of statistical analyses, monkeypox virus neutralizing antibodies were grouped as follows: low (1:20–1:40), medium (1:80), and high (1:160–1:320); people with titers ≤ 1:10 were excluded due to the very low number of people included in this group (n = 2 with titer = 1:10, n = 0 with titer < 1:10). The differences in monkeypox virus-neutralizing antibody titers depending on clinical and virological characteristics were evaluated by the Chi-square, Mann–Whitney, and Kruskal–Wallis’s tests. Post-hoc analyses for significant comparisons were assessed with the Dwass test. The comparisons and corresponding *p*-values, considering Bonferroni’s correction, are reported with box and bar plots. Analyses were performed considering either all samples (cutaneous, anal, oropharyngeal, plasma, and seminal fluids grouped), swabs (cutaneous, anal, and oropharyngeal grouped), or specific samples. For the analyses with all samples or swabs pooled, the lowest cycle threshold (Ct) was considered; association between Ct values and neutralizing antibodies was done considering Ct either as a continuous variable or Ct stratified according to the median and interquartile values of each sample. Considered virological characteristics were Cts at baseline, days with positive monkeypox virus (total days from baseline to last viral detection), and having positive/negative specific samples (anal, oropharyngeal, plasma, and seminal fluids). Univariable linear regression was used to assess the association between monkeypox virus neutralizing antibody titers (categorical independent variable) and the logarithm of days with positive monkeypox virus (dependent variable). The logarithm was used to fit the assumption of normality for the linear regression model. For the purpose of analyses regarding the virologic aspects of the monkeypox virus, the time of mpox diagnosis by means of PCR was defined as baseline.

A two-sided probability value (*p*-value) < 0.05 was considered statistically significant. Analyses were done using R Statistical Software, version 4.2.3 (R Foundation for Statistical Computing, Vienna, Austria). Background research was conducted up to April 2024 on PubMed and Scopus, including key terms “mpox” or “monkeypox” or “MPXV” and “serology” or “antibodies” or “immunity” or “immune” or “serologic” or “neutralizing”.

## 3. Results

### 3.1. Study Population and Characteristics of Mpox Infection

Overall, 95 men who have sex with men (MSM) with previous mpox diagnosis were included in this study. The median age was 39.4 years (interquartile, IQR = 35.4–44.7); 33 (34.7%) were HIV pre-exposure prophylaxis (PrEP) users, and 50 (52.6%) were PLWH. Previous smallpox vaccination in their youth, as per national smallpox immunization programs, was reported by 16 (16.8%). Among PLWH, HIV-RNA was <50 copies/mL in 44 (89.8%), and the median CD4^+^ T lymphocyte count was 690 cells/microL (IQR = 559–1005) at the time of mpox infection. Regarding the clinical characteristics of mpox, the median number of lesions was 5 (IQR = 3–10). The median number of days from the onset of symptoms of mpox to complete resolution was 18 days (IQR = 13–24). Overall, 71/78 (91%) people had positive cutaneous swabs, 54/69 (78%) anal, 59/87 (68%) oropharyngeal, 55/72 (76%) plasma, and 15/30 (50%) seminal fluids at baseline. The median Ct values of cutaneous swabs were 20 (IQR = 17–24), of anal swabs 23 (IQR = 18–28), of oropharyngeal swabs 29 (IQR = 27–33), of plasma 34 (IQR = 33–35), and of seminal fluids 32 (IQR = 29–35). The median days with detection of the monkeypox virus in cutaneous swabs were 16 (IQR = 9–19), in anal swabs 12 (IQR = 7–18), in oropharyngeal swabs 14 (IQR = 9–18), in blood 9 (IQR = 7–13), and in seminal fluids 8 (IQR = 7–15). When considering all swabs (cutaneous, oropharyngeal, and anal, all grouped together), the median baseline Ct was 19 (IQR = 16–23) and the median days of detection of monkeypox virus were 18 (IQR = 13–23). When considering all analyzed samples (cutaneous, anal, oropharyngeal, plasma, and seminal fluids all grouped together), the median baseline Ct was 19 (IQR = 16–23) and the median days of detection of monkeypox virus were 19 (IQR = 14–24).

### 3.2. Assessment of Neutralizing Antibodies Titers

Plaque reduction neutralization test showed the following monkeypox virus neutralizing antibody titers: in 2/95 (2.1%) individuals were 1:10, in 6/95 (6.3%) were 1:20, in 20/95 (21.1%) were 1:40, in 29/95 (30.5%) were 1:80, in 21/95 (22.1%) were 1:160, and in 17/95 (17.9%) were 1:320. Complete PRNT results, also according to HIV status, are presented in Table 1.

All people developed detectable neutralizing antibodies (≥1:10) following mpox infection.

### 3.3. Association between Characteristics of Mpox Infection and Antibodies Titers

**Figure 1:**
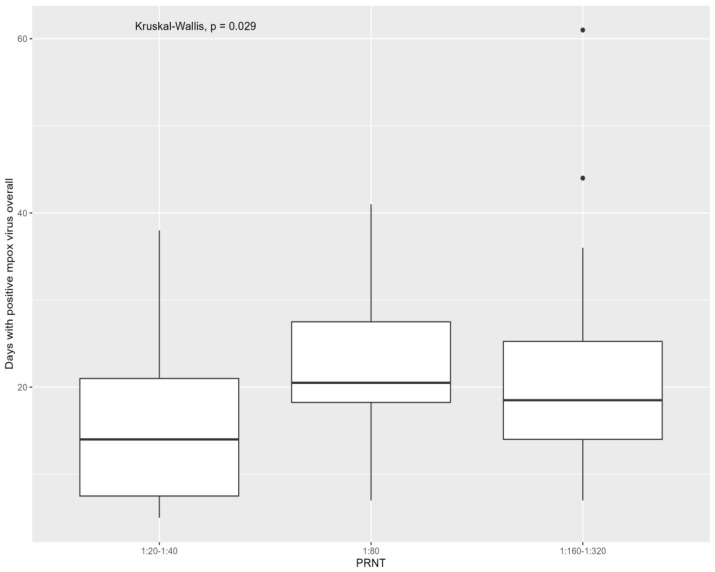
Distribution of mpox neutralizing antibodies at six months from infection according to days with positive mpox virus (all samples grouped).

**Figure 2:**
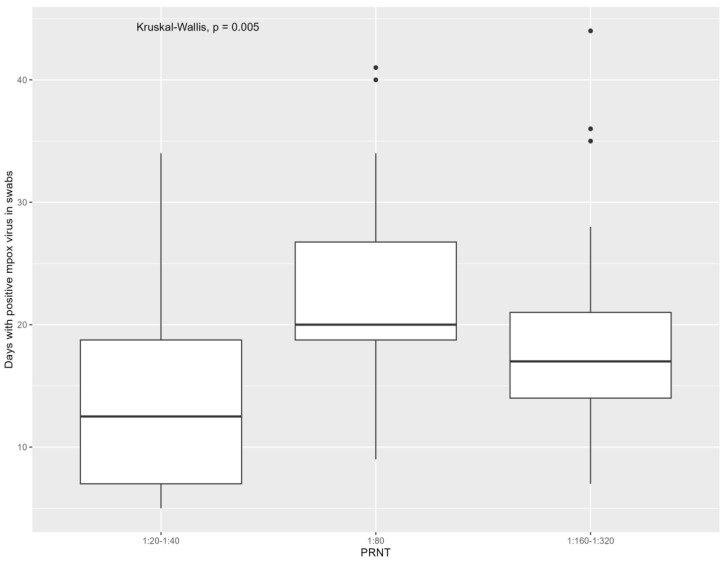
Distribution of mpox neutralizing antibodies at six months from infection according to days with positive mpox virus (only swabs grouped).

Referring to the virological characteristics of mpox, people who were positive for monkeypox virus for more days had higher levels of neutralizing antibodies when considering both samples [1:20–1:40: 14 days (IQR = 7–21), 1:80: 20 days (IQR = 18–27), 1:160–1:320: 18 days (IQR = 14–25), overall *p*: 0.029; due to the significant difference in 1:80 versus 1:20–1:40 (*p*: 0.01)] and all swabs [1:20–1:40: 12 days (IQR = 7–19), 1:80: 20 days (IQR = 19–27), 1:160–1:320: 17 days (IQR = 14–21), overall *p*: 0.01; due to significant difference in 1:80 versus 1:20–1:40 (*p*: 0.01)] grouped. The association of overall days of mpox detection with monkeypox virus neutralizing antibodies is presented in Figure 1 and Figure 2.

No difference in terms of neutralizing antibody titers was found when considering the median days of monkeypox virus detection in specific samples (anal swabs *p*: 0.27, cutaneous swabs *p*: 0.33, oropharyngeal swabs *p*: 0.37, plasma *p*: 0.66, and seminal fluids *p*: 0.93). At univariable linear regression, the logarithm of the total days of monkeypox virus detection was found to directly correlate with the neutralizing antibody titers both when considering all samples (1:80 versus 1:20–1:40, β: 0.435, *p*: 0.008; 1:160–1:320 versus 1:20–1:40, β: 0.27, *p*: 0.06) or all swabs (1:80 versus 1:20–1:40, β: 0.154, *p* < 0.001; 1:160–1:320 versus 1:20–1:40, β: 0.14, *p*: 0.03) grouped.

**Figure 3:**
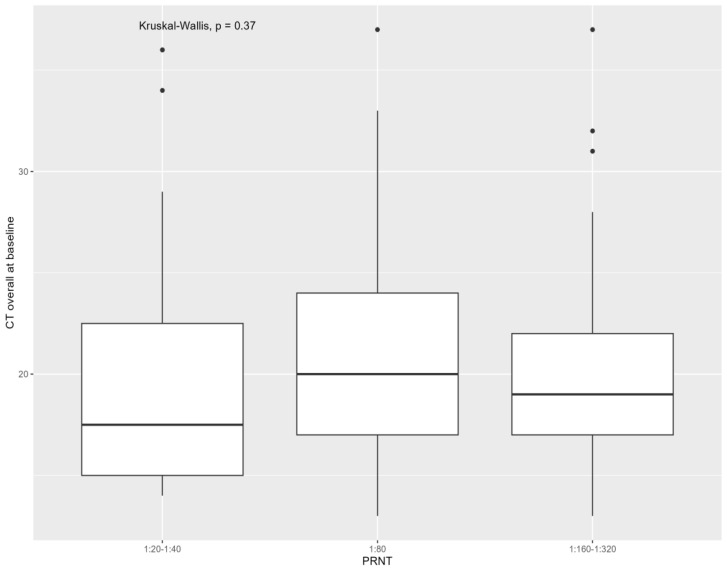
Cycle threshold (Ct) of monkeypox virus (considering cutaneous swabs, anal swabs, oropharyngeal swabs, plasma, and seminal fluids) at time of mpox diagnosis according to monkeypox virus neutralizing antibodies assessed with plaque reduction neutralization test (PRNT) at 6 months from mpox. Note: The Kruskal–Wallis test showed no significant association between the cycle threshold and the clinical duration of mpox (*p*: 0.37).

**Figure 4:**
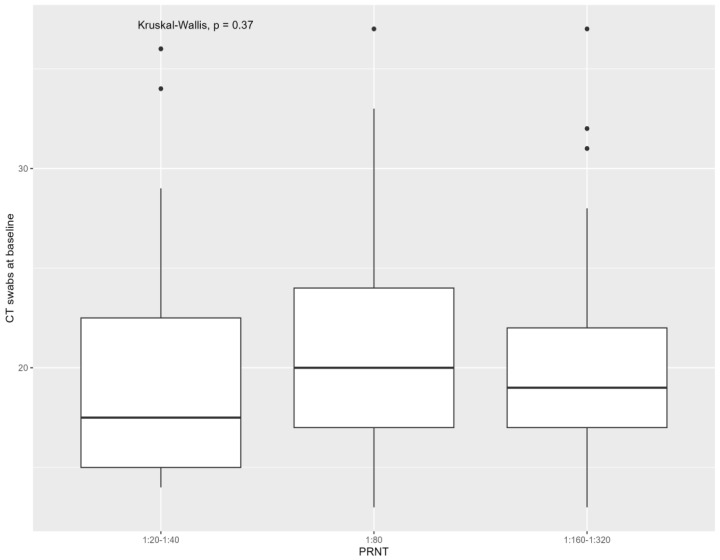
Cycle threshold (Ct) of monkeypox virus (considering cutaneous, anal, and oropharyngeal swabs) at time of mpox diagnosis according to monkeypox virus neutralizing antibodies assessed with plaque reduction neutralization test (PRNT) at 6 months from mpox. Note. Kruskal–Wallis test showed no significant association between the cycle threshold and the clinical duration of mpox (*p*: 0.37).

The median baseline Ct considering all samples (*p*: 0.370) and all swabs (*p*: 0.37) was not different between the three neutralizing antibody groups. The association of baseline CT with monkeypox virus neutralizing antibodies is presented in Figure 3 and Figure 4. No difference in antibody titers was found between people with positive baseline anal swabs (*p*: 0.98), oropharyngeal (*p*: 0.91), plasma (*p*: 0.99), or seminal fluids (*p*: 0.29) compared with those who were negative at baseline. Considering samples positive at baseline, the median Ct was not different between the three neutralizing antibody groups, referring to anal swabs (*p*: 0.43), cutaneous swabs (*p*: 0.28), plasma (*p*: 0.88), and seminal fluids (*p*: 0.61) (continuous variable). The median Ct was also not different in the three antibody groups considering all samples (*p*: 0.47), all swabs (*p*: 0.47), anal swabs (*p*: 0.81), cutaneous swabs (*p*: 0.38), oropharyngeal swabs (*p*: 0.11), plasma (*p*: 0.56), and seminal fluids (*p*: 0.41) (analyzed with stratified Ct).

Considering the clinical characteristics of mpox, we did not identify any characteristics associated with different monkeypox virus-neutralizing antibody titers. For instance, no difference in the proportion of people with low, medium, or high neutralizing antibody titers was found in relation to the presence of fever (*p*: 0.27), lymphadenopathy (*p*: 0.46), diffuse rash (*p*: 0.17), pharyngitis (*p*: 0.40), or proctitis (*p*: 0.27). Regarding the distribution of mpox related lesions, we did not identify any difference regarding the presence of cutaneous lesions (*p*: 0.70), oral lesions (*p*: 0.61), genital lesions (*p*: 0.92), or anal lesions (*p*: 0.39). No difference in the median number of lesions was found between the low, medium, and high antibody titers groups (*p*: 0.78). The median days with the presence of mpox clinical symptoms were not different between the three groups of neutralizing antibody titers (*p*: 0.10).

No differences in median age were found between the monkeypox virus-neutralizing antibody groups (*p*: 0.14). Monkeypox virus neutralizing antibodies were found to be associated with the previous smallpox vaccination status (*p*: 0.03) and to be living with HIV (*p*: 0.04), but not with the CD4^+^ T lymphocyte levels at the time of mpox infection diagnosis (*p*: 0.80).

## 4. Discussion

In this cohort of MSM diagnosed with mpox, all developed monkeypox virus-neutralizing antibodies at six months from infection, also among PLWH with a good immune-virologic status. Higher antibody titers were found among people with longer detection of the monkeypox virus in the body, both when considering all samples (including lesions, oropharynx, anal, plasma, and seminal fluids) or only swabs (including lesions, oropharynx, and anal). In details, people with previous mpox infection with neutralizing antibody titers greater than 1:80 had monkeypox virus detected for more days than people with titers less than 1:80. Though, when considering single sample sites, we found no specific association between the overall days of monkeypox virus detection in a specific anatomical site and the neutralizing antibodies.

All considered clinical characteristics of mpox, including duration of mpox symptoms, were found to be not predictive of higher or lower neutralizing antibody titers. For instance, indicators of severe mpox, which include the median number of lesions and atypical mpox symptoms like the presence of rectal or pharyngeal involvement, were not found to be associated with monkeypox virus-neutralizing antibodies.

Possibly, we identified as a predictive factor of antibody response at high or low levels the total days of monkeypox virus detection because virus presence in the body is the main driver of the immunologic response as opposed to clinical aspects of infection. As previously reported in retrospective and prospective cohort studies on people diagnosed with mpox, the viral dynamics of the monkeypox virus are heterogeneous among cases, showing different viral loads and variable positive sites, which could be linked to the way of exposure or unpredictable viral dynamics [4,20,22,23]. Indeed, in this very study, we observed a diversity in terms of the percentage of people showing specific positive sample sites at the time of mpox infection diagnosis, with the vast majority of individuals having positive cutaneous swabs and far fewer people with positive seminal fluids and plasma. Moreover, when evaluating the overall duration of detection of the monkeypox virus at the specific sample site, we observed strong diversity. In detail, the overall duration was longer in cutaneous swabs and significantly shorter in blood and seminal fluid samples, which is also in line with other previous virologic studies [20]. The kinetics of monkeypox virus detection were indeed heterogeneous among people and when considering specific samples, which could be linked to different clinical presentations or sites of infection, along with differences in disease severity and immunological status. Therefore, we believe that the analysis, which encompasses all different tested sites together, better reflects the true overall days of monkeypox virus presence in the body, which we hypothesize is the main driver of the immunological response.

We acknowledge that this retrospective study included samples that were not collected prospectively. However, we believe that the high number of included specimens over the course of the follow-up at different anatomical sites likely mitigates this limitation of the study. Moreover, the number of included individuals is a study limitation, which might limit the results applicability to the overall population. As of now, another question is whether neutralizing immune responses against mumps will be long-lasting. As a matter of fact, there is undeniable evidence of long-living humoral immunity against smallpox, induced by vaccination or a naturally occurring disease, but there is still little to no evidence on the duration of immunity elicited by mpox. Indeed, a single timepoint of neutralizing antibody evaluation was considered in this study, without the opportunity to evaluate the waning of immunity over time. Further assessment of multiple time points over time following the recovery of the infection could provide further valuable insight into the kinetics of immunity to monkeypox virus infection. Moreover, the plaque reduction neutralization assay, which we used, might also be prone to some limitations. While acknowledging the potential influence of complement on the described serum neutralization assay, all tested serum samples underwent heat treatment (56 °C for 30 min) to mitigate any interference, as detailed in the methods section [24,25].

Identification of predictive factors of a higher or lower immunologic response following mpox is needed to grant further insight on mpox pathogenesis and immunological signature. From a public health perspective, these findings should also be taken into account, given the recent evidence of mpox re-infections, which pose a global health threat to effective infection containment [17]. However, we note that monkeypox virus-neutralizing antibody protective thresholds are unknown, limiting the applicability of these study findings in clinical practice. For instance, the identified threshold of monkeypox neutralizing antibodies (i.e., 1:80), which can be applied only to the specific plaque reduction neutralization test we designed and described, represents a possible cut-off to describe a more robust immunologic response, although other immunologic factors, such as cellular immunity, likely contribute to protection from mpox infection. Recently, the Mpox Severity Scoring System (MPOX SSS) has been suggested as a possible clinical tool to predict the severity of infection and has been used to describe some international cohorts in terms of the clinical presentation of disease over time [17,26]. Combining clinical factors, aimed at assessing the severity of disease, with virologic factors, as predictors of possible neutralizing antibodies serologic response, could be useful to provide a better framework on the disease’s natural history and the risk of re-infections.

The global response to the mpox outbreak has been a testament to the importance of international collaboration, such as the examples of the SHARE NET network, and the need for agile, evidence-based public health policies [1,17]. The challenges encountered in curbing the spread of the virus have underscored the critical importance of surveillance, rapid response mechanisms, and community engagement in managing health crises. As the world continues to navigate the repercussions of the mpox outbreak, the lessons learned will be invaluable in strengthening global health security and preparedness for future infectious disease threats.

## 5. Conclusions

In conclusion, this study found that the overall days of monkeypox virus detection in the body, irrespective of the viral loads or the clinical manifestations of infection, directly correlated with monkeypox virus neutralizing antibodies at six months from infection.

## Figures and Tables

**Table 1:** Monkeypox virus neutralizing antibodies assessed with plaque reduction neutralization test (PRNT) test results at 6 months from mpox according to HIV status.

PRNT	Overall N = 95	Living without HIV N = 45	Living with HIV N = 50
1:10	2 (2.11%)	1 (2.22%)	1 (2.00%)
1:20	6 (6.32%)	0 (0.00%)	6 (12.0%)
1:40	20 (21.1%)	7 (15.6%)	13 (26.0%)
1:80	29 (30.5%)	17 (37.8%)	12 (24.0%)
1:160	21 (22.1%)	13 (28.9%)	8 (16.0%)
1:320	17 (17.9%)	7 (15.6%)	10 (20.0%)

## Data Availability

The data that support the findings of this study are available on request from the corresponding author. The data are not publicly available due to privacy or ethical restrictions.
